# Compositional and Interface Engineering of Organic-Inorganic Lead Halide Perovskite Solar Cells

**DOI:** 10.1016/j.isci.2020.101359

**Published:** 2020-07-10

**Authors:** Haizhou Lu, Anurag Krishna, Shaik M. Zakeeruddin, Michael Grätzel, Anders Hagfeldt

**Affiliations:** 1 Laboratory of Photomolecular Science, Institute of Chemical Sciences Engineering, École Polytechnique Fédérale de Lausanne (EPFL), 1015 Lausanne, Switzerland; 2 Laboratory of Photonics and Interfaces, Institute of Chemical Sciences and Engineering, École Polytechnique Fédérale de Lausanne (EPFL), 1015 Lausanne, Switzerland

**Keywords:** Applied Chemistry, Materials Chemistry, Energy Materials

## Abstract

Power conversion efficiency (PCE) of the perovskite solar cells (PSCs) has remarkably been increased from 3.1% to 25.2%. The fast expansion of the PSCs has been along with the development of compositional and interface engineering, which has been playing a critical role. For the PSCs with record high-efficiency and stability, the perovskite absorber layer has been changed from the initial MAPbI_3_- to FAPbI_3_-based compositions. Owing to the enormous engineering works, perovskite absorber layers with monolithic grains could be achieved, in which the interior defects are negligible compared with the surface defects. Therefore, interface engineering, which can passivate the surface defects and/or isolate the perovskite from the environmental moistures, has been playing a more and more important role to further boost the PCE and stability of the PSCs. Herein, a compact review study of the compositional and interface engineering is presented and promising strategies and directions of the PSCs are discussed.

## Highlights

• Perovskite compositional engineering from MAPbI_3_ to FAPbI_3_
• Stabilization of α-FAPbI_3_
• Passivation strategies regarding different types of defects
• Future perspectives of efficient and stable PSCs

## Graphical Abstract

**Figure:**
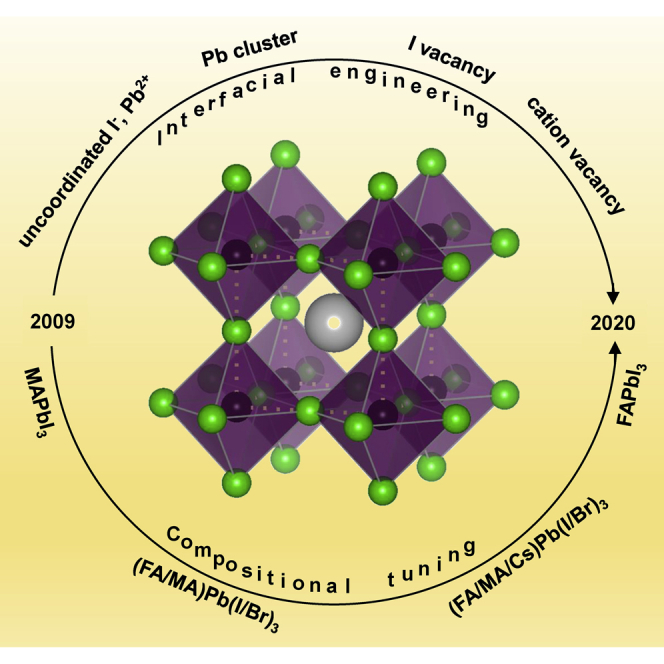

## Introduction

Organic-inorganic metal halide perovskite solar cells (PSCs), as emerging photovoltaics, have attracted a lot of attention since the first PSC reported in 2009 (Kojima et al., 2009). Within a decade, significant progress has been achieved; for example, power conversion efficiency (PCE) has been increased from 3.1% to 25.2% (www.nrel.gov/pv/cell-efficiency.html), which makes PSCs exceed other thin-film solar cells and even the market leader polycrystalline silicon solar cells. In the meantime, the PSCs have been reported to work under operational conditions for over 1,000 h with additive engineering (Bai et al., 2019; Zheng et al., 2020) or using poly(3-hexylthiophene) hole-transporter layer (Jung et al., 2019), even though the operational stability test was carried out at 25°C instead of 60°C, the standard test condition. These achievements are mainly attributed to the unique properties of the perovskites, such as high absorption coefficients, low non-radiative recombination rate, high carrier mobility, and long carrier diffusion length (Xing et al., 2013; Stranks et al., 2013).

**Figure 1:**
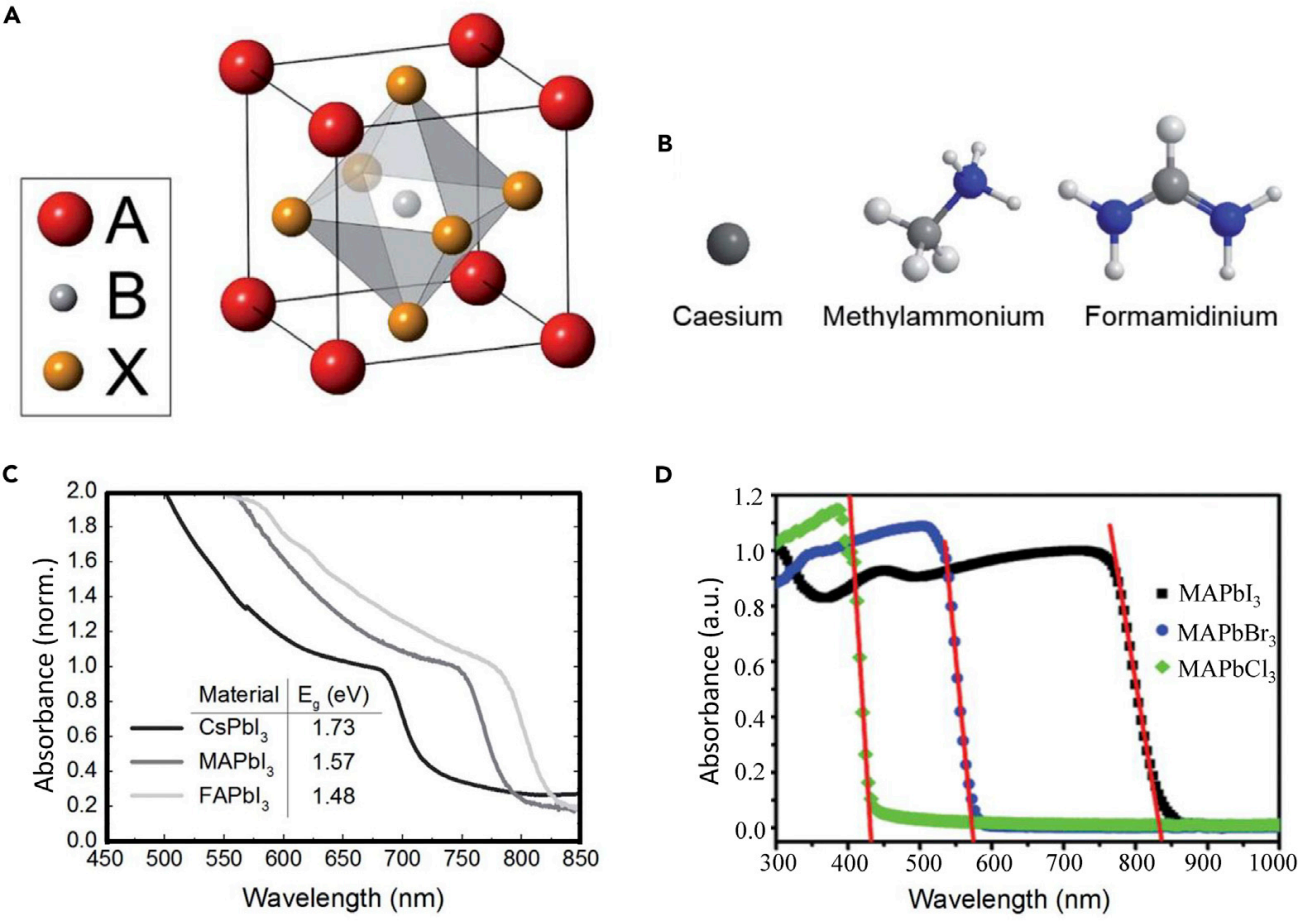
Perovskite Crystal Structure and Band-gaps (A) The ABX_3_ perovskite crystal structure. (B) The atomic structure of the three A site cations including Cs, MA, and FA. (C and D) (C) UV-vis spectra of the APbI_3_ perovskites, where A is Cs, MA, or FA. (D) UV-vis spectra of the MAPbX_3_, where X is I, Br, or Cl. (A–C) Reproduced with permission (Eperon et al., 2014). Copyright 2014, Royal Society of Chemistry. (D) Reproduced with permission (Liu et al., 2015). Copyright 2015, Wiley-VCH.

Metal halide perovskites have the formula ABX_3_, where A is a monovalent cation, B is a divalent metal cation, and X is a halide anion. The crystal structure of the widely studied CH_3_NH_3_PbI_3_ is illustrated in Figure 1A. In principle, any molecular cation could be used, once there is enough space to fit it within the cavity. The formation of the 3D perovskite network is determined by the Goldschmidt tolerance factor [$t=\frac{r_{A}+\;r_{X}}{\sqrt{2}\;\left(r_{B}+\;r_{X}\right)}$, where ris the ionic radius of each component (A, B or X in ABX_3_)]. Based on this, different cations like Cs, methylammonium (MA), and formamidinium (FA) (shown in Figure 1B) and anions like I, Br, and Cl, and their combinations, have been explored in the past few years. By tuning the cations and/or anions of the A, B, and X sites, various band gaps as shown in Figures 1C and 1D could be achieved for applications such as full-color perovskite light-emitting diodes (Sutherland et al., 2016) and tandem PSCs (McMeekin et al., 2016).

Since the first protocol of MAPbI_3_- and MAPbBr_3_-based PSCs reported in 2009 (Kojima et al., 2009), there have been enormous research works focusing on the perovskite compositional engineering to improve the PCE as well as stability of the PSCs (Jeon et al., 2015; Saliba et al., 2016a, 2016b; Turren-Cruz et al., 2018). Until now, the most commonly used perovskite composition has been changed from the initial MAPbI_3_ to the so-called triple cation MA_x_FA_0.95-x_Cs_0.05_Pb(I_1-y_Br_y_)_3_ perovskite, and even toward the pure FAPbI_3_ perovskite for the record high-efficiency PSCs reported recently (Jiang et al., 2019; Kim et al., 2019; Min et al., 2019). The latter trend is due mainly to FA being thermally more stable than MA, and the band gap of FAPbI_3_ is closer to the Shockley-Queisser optimum compared with that of MAPbI_3_ and to compositions with mixed halides. Thus, FAPbI_3_-based PSCs could potentially have better stability and higher efficiency than MAPbI_3_-based ones. Furthermore, the carrier diffusion length of the FAPbI_3_ perovskite was reported to be significantly longer than that of MAPbI_3_, making a few hundred nanometer-thin FAPbI_3_ layer suitable for even planar structure-based PSCs (Eperon et al., 2014). Although the FAPbI_3_ perovskite shows obvious advantages, it is practically difficult to deposit highly crystallized pure α-phase FAPbI_3_ films. Hence, there have been many reported compositional engineering works that compromised the optimum band gap with the perovskite crystallinity and/or stability. After years of efforts, recently highly efficient and stable PSCs have been achieved with a perovskite composition that is very close to pure FAPbI_3_ (Kim et al., 2019; Min et al., 2019). We note that this review focuses only on the seminal works done on the organic-inorganic lead halide perovskites.

In addition to the compositional engineering, interface engineering has also been playing an important role toward the highly efficient and stable PSCs. We know that a high density level of defects is likely to exist inside the bulk and on the surface of the perovskite film when it is fabricated through a solution process. These defects, including under-coordinated ions and dangling bonds, could result in non-radiative charge recombination and possible degradations of the PSCs. Thus, suppressing these defects is necessary to further improve the efficiency and stability of PSCs. Benefited from the compositional engineering and optimized perovskite deposition methods, high-quality perovskite films with monolithic grains have been achieved, in which the interior defects are negligible compared with the surface defects. Therefore, interface engineering has become more and more important for further improving the performance of the PSCs. Especially, in case of the organic-inorganic lead halide perovskites, the nature of soft organic cation makes the interface engineering more important than the case of thermally stable all-inorganic perovskites. We note that interface engineering has been adopted for all the record-efficiency PSCs reported recently (Jiang et al., 2019; Kim et al., 2019; Min et al., 2019). There have been several reports of molecules with functional groups, including carbonyl, cyano, and amine units, to neutralize the surface charges or coordinate with the surface point defects to annihilate the corresponding electronic traps. There are also some reports on separating the 3D bulk perovskite layer from the electron/hole-transporter layer with a hydrophobic 2D perovskite layer (Cho et al., 2017; Liu et al., 2019) or graphene-based monolayers (Bi et al., 2017; Wang et al., 2019), which improves substantially the operational stability of the PSCs.

In this review, we will first discuss the strategies of perovskite compositional engineering from MAPbI_3_ to FAPbI_3_ perovskite based on the tuning of A cations and/or X anions, as well as the relationships between perovskite composition and the performance as well as the stability of the PSCs. We will then discuss the promising interface engineering toward highly efficient and stable PSCs. In the end, we further discuss some directions for the development of compositional and interface engineering of perovskite layers and devices.

## Perovskite Compositional Engineering

### Methylammonium (MA)-Based PSCs

In 2009, MA-based PSCs were first demonstrated as liquid junction solar cells, which showed poor stability due to the liquid-type electrode (Kojima et al., 2009). Three years later, Kim et al. and Lee et al. reported MAPbI_3_-based solid-state mesoscopic heterojunction PSCs independently (Kim et al., 2012; Lee et al., 2012), which showed a dramatically improved device stability compared with that of the previous liquid junction solar cells. Since then, PSCs have been hot research topics, and the MAPbI_3_ perovskite has been used as a standard perovskite composition. MAPbI_3_ has a tetragonal phase at room temperature. The PbI_6_ octahedra in MAPbI_3_ are corner connected, and the MA^+^ cations occupy the octahedra interstices, as shown in Figure 1A. Importantly, MA-based perovskite films could be achieved by a simple solution spin coating process, which gives it an advantage over the other thin-film photovoltaic techniques.

**Figure 2:**
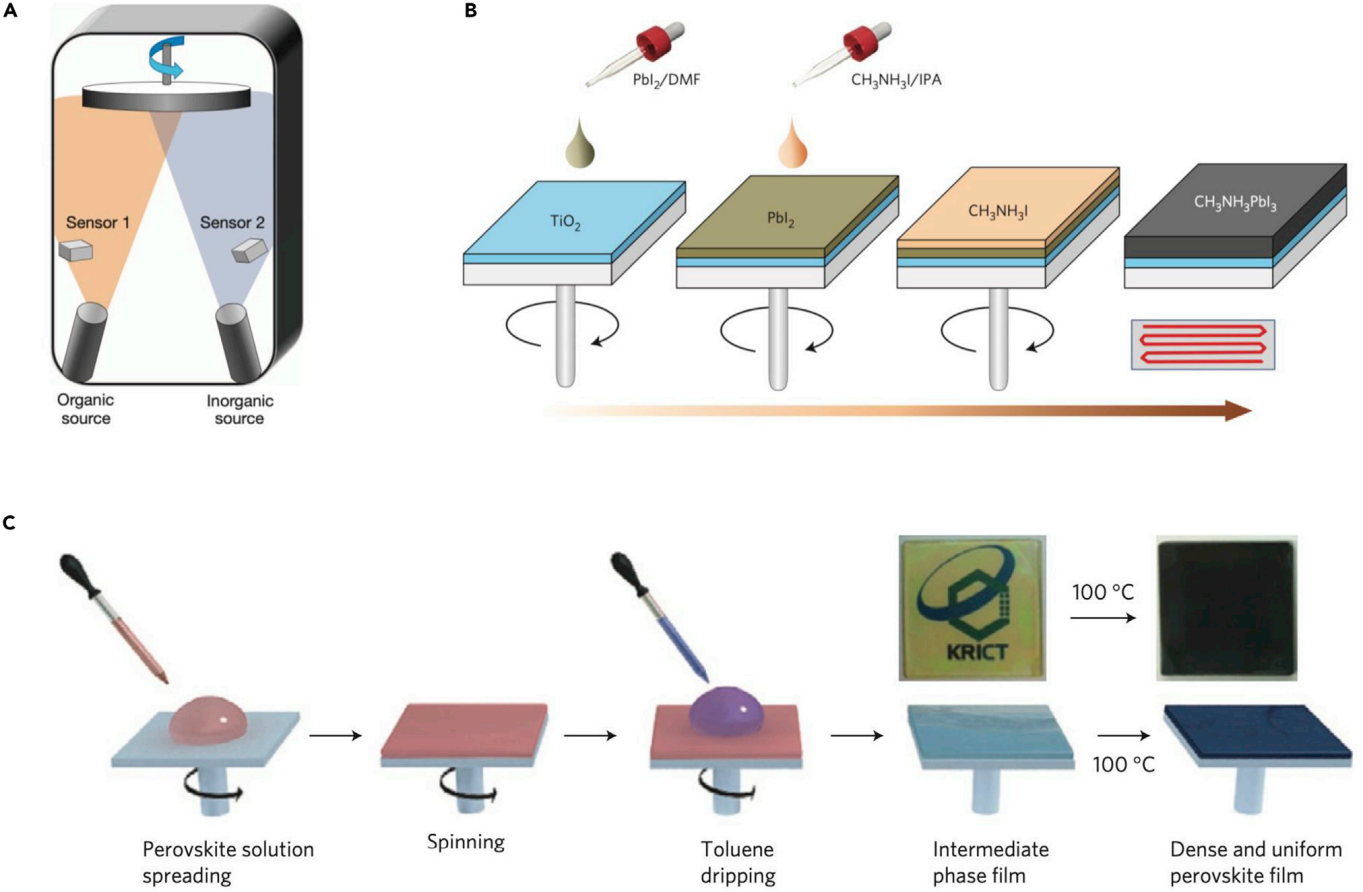
Depostion Methods of Perovskite (A) Dual source thermal evaporation system for depositing the perovskite absorbers. The organic source is MAI, and the inorganic source is PbCl_2_. Reproduced with permission (Liu et al., 2013). Copyright 2013, Nature Publishing Group. (B) A demonstration of the two-step deposition of MAPbI_3_ perovskite. Reproduced with permission (Im et al., 2014). Copyright 2014, Nature Publishing Group. (C) Anti-solvent engineering procedure of preparing the uniform and dense perovskite film. Reproduced with permission (Jeon et al., 2014). Copyright 2014, Nature Publishing Group.

At the very beginning, the PCE of the MA-based PSCs was below 10%, which is mainly due to the poor crystallinity and/or morphology of the spin-coated perovskite films. To improve the quality of the MA-based perovskite films, many fabrication methods as shown in Figure 2, including thermal evaporation (Liu et al., 2013), sequential deposition (Burschka et al., 2013; Im et al., 2014), and anti-solvent dipping (Jeon et al., 2014) were developed subsequently. Surprisingly, the PCE of the MAPbI_3_-based PSCs had already been boosted to ∼17% by 2014. Moreover, halide mixing was also found to be able to greatly improve the stability of the MA-based PSCs. Jeon et al. (2014) and Noh et al. (2013) reported that a substitution of 10–15 mol % Br^−^ for I^−^ in MAPbI_3_ can improve the stability and efficiency of the PSCs. A certified efficiency of 16.2% was achieved for the MAPb(I_1-x_Br_x_)_3_ (x = 0.1–0.15) composition-based PSCs (Jeon et al., 2014). Interestingly, although Cl^−^ does not substitute I^−^ of MAPbI_3_ it acts as a crystallization aid improving the carrier diffusion length (Stranks et al., 2013) and the efficiency of the corresponding PSCs (Liu et al., 2013). We also note that the substitution of Br^−^ for I^−^ in MAPbI_3_ can tune the band gap between 1.53 eV and 2.97 eV (Liu et al., 2015) as shown in Figure 1D, which makes it possible to have colorful PSCs (Noh et al., 2013).

Although both the efficiency and stability of the MA-based PSCs have been improved significantly, especially a PCE of around 20% was achieved with molecular controlled intermediates (Ahn et al., 2015; Cao et al., 2016), it is still far from the final commercializations. Since 2015, more and more attention has been paid to the FA-based PSCs, which could potentially be more stable and efficient than the MA-based ones.

### Formamidinium (FA)-Based PSCs

**Figure 3:**
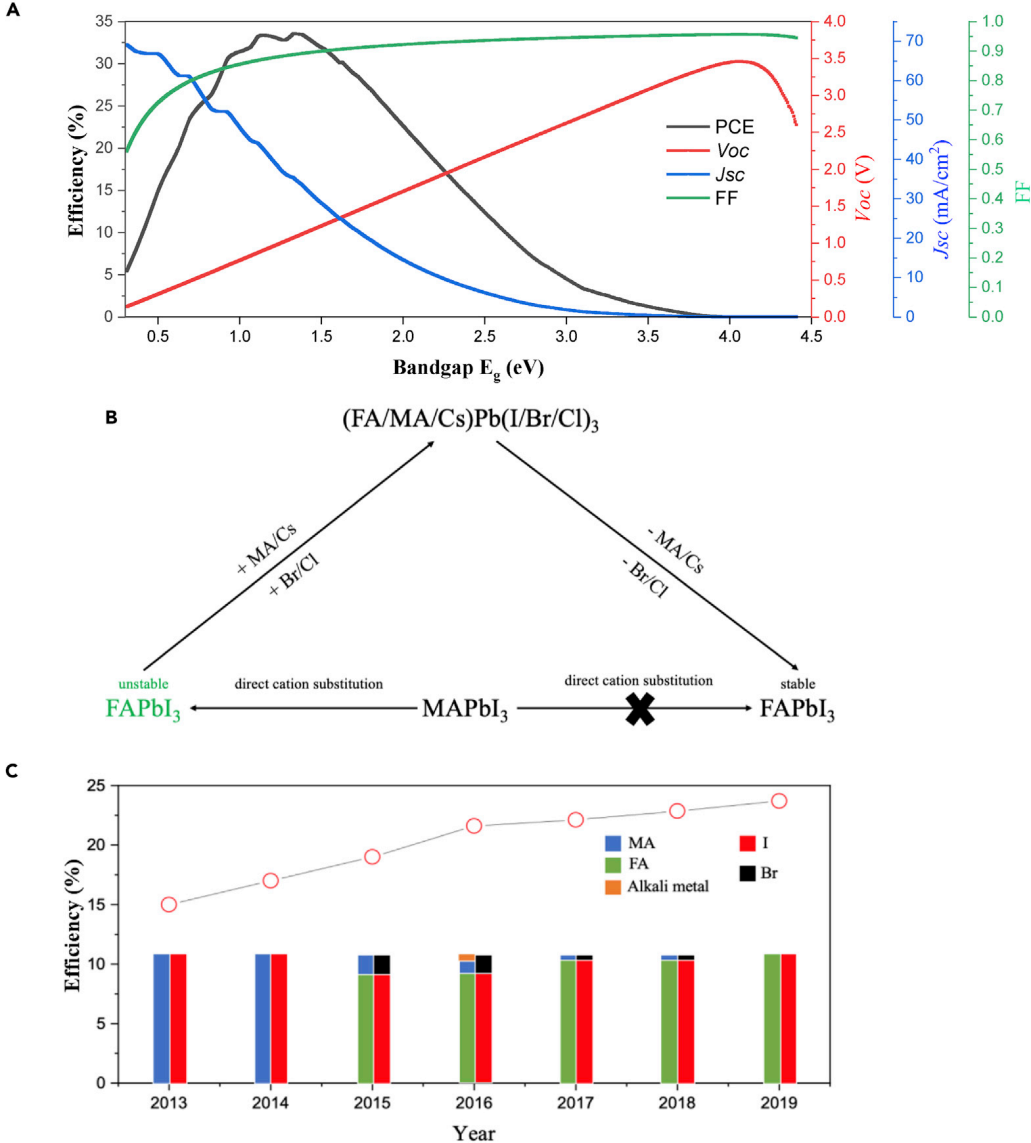
Perovskite Compositional Change with Time (A) Shockley-Queisser limit power conversion efficiency (PCE), open circuit voltage (V_OC_), short circuit current density (J_SC_), and fill factor (FF) as a function of band gap. (B) A simplified diagram illustrating the direction of compositional change of the perovskite absorber. (C) Comparison of the efficiency and perovskite compositions of PSCs from literature (Saliba et al., 2016a; Min et al., 2019; Liu et al., 2013; Im et al., 2014; Yang et al., 2015, 2017; Jeon et al., 2018).

MAPbI_3_ does not absorb as much of the solar spectrum as an optimum single-junction solar cell. This is due to its band gap of ∼1.55 eV, whereas the Shockley-Queisser optimum would be 1.1–1.4 eV, as shown in Figure 3A. Replacing the MA with a slightly bigger size FA could reduce the band gap of the perovskite to ∼1.48 eV (Figure 1C), approaching the Shockley-Queisser optimum. Therefore, FAPbI_3_-based PSCs could potentially have higher PCE than the MAPbI_3_-based ones due to the increased absorption. Regarding the stability issue, FA is much more stable than the volatile MA cation. Furthermore, FAPbI_3_ has a Goldschmidt tolerance factor close to 1, which suggests an ideal crystal structure. Therefore, FAPbI_3_ could be an interesting perovskite composition toward the final commercialization of the PSCs.

FAPbI_3_-based PSCs were first reported by Eperon et al. (2014), and an efficiency of 14.2% was achieved at that time. It was found that FAPbI_3_ was thermally more stable than MAPbI_3_, especially under heat stress, which is very important for the long-term operational stability. Unfortunately, the non-photoactive δ-phase FAPbI_3_ is the most stable phase at room temperature. Phase stability has been a main concern for the FAPbI_3_ perovskite compositions. It also has been a challenge to fabricate highly crystallized pure α-phase FAPbI_3_ due to its relatively high thermodynamic α-phase transition temperature. To improve the crystallinity and stabilize the α-phase FAPbI_3_, multi-cation and/or halide mixing strategies have been developed toward the highly efficient and stable FAPbI_3_-based PSCs.

### (FA_1-x_MA_x_)Pb(I_1-y_Br_y_)_3_-Based PSCs

FAPbI_3_ has a broader absorption spectrum than MAPbI_3_, which makes FAPbI_3_ a promising candidate for the PSCs. However, the PCE of the FAPbI_3_-based PSCs did not show any significant advance over MAPbI_3_-based ones at the beginning (Eperon et al., 2014; Lee et al., 2014), which is mainly due to the poor quality of the FAPbI_3_ films. The PCE (*P*) is determined by the product of current density *J*_*SC*_, open-circuit voltage *V*_*OC*_, and fill factor (*FF*) $\left(P=J_{SC}*V_{OC}*FF\right)$. In the initial studies, the *J*_*SC*_ (23.3 mA/cm^2^) achieved for the FA-based PSCs is close to the Shockley-Queisser limit, whereas the *V*_*OC*_ (0.94 V) and FF (0.65) are still lagging (Eperon et al., 2014), which prevented the FAPbI_3_-based PSCs from achieving efficiencies over 20%. Thus, further engineering work is necessary to improve the quality of FAPbI_3_ films.

To tackle the issue of the poor crystallinity and morphology as well as the δ-phase impurities of the FAPbI_3_ film, Pallet et al. (2014) reported a strategy using a mixture of MA and FA cations. Surprisingly, the mixed cation perovskite composition (FA_1-x_MA_x_PbI_3_) remained the broad absorption range of the FAPbI_3_, but the phase impurities were eliminated and the crystallinity was much improved. Consequently, a superior PCE of 15% was achieved for the FA_1-x_MA_x_PbI_3_-based PSCs. Later, et al. (Yang et al. (2015) reported that additional Br^−^ halides mixed composition could further boost both the efficiency and stability of the PSCs. It was found that additional Br^−^ halides could contribute to the crystallization process of the perovskite film, which resulted in greatly improved morphology and stability of the final perovskite composition (FA_1-x_MA_x_Pb(I_1-y_Br_y_)_3_). The highest reported efficiency by Yang et al. (2015) was over 20%, which was due to the improved *V*_*OC*_ and FF. In 2016, Bi et al. reported a certified efficiency of 21% using a polymer-templated nucleation method (Bi et al., 2016). Yang et al. (2017) further increased the efficiency of the FA_1-x_MA_x_Pb(I_1-y_Br_y_)_3_-based PCSs to a certified value of 22.1% using a two-step deposition method. Similarly, Jiang et al. (2017) introduced MACl into the FA_1-x_MA_x_Pb(I_1-y_Br_y_)_3_ perovskite composition, where MACl could be re-evaporated during the annealing process, and a certified efficiency of 21.6% was reported for planar structure PSCs. In 2018, a certified efficiency of 22.85% was reported by Jeon et al. (2018) using a fluorene-terminated hole-transporting material (HTM). In all, the FA_1-x_MA_x_Pb(I_1-y_Br_y_)_3_ perovskite composition has shown a fast and promising development of the FAPbI_3_-based PSCs.

### Alkali Metal-Doped (FA_1-x_MA_x_)Pb(I_1-y_Br_y_)_3_-Based PSCs

Significant progress has been achieved for the FA-based PSCs by using the FA_1-x_MA_x_Pb(I_1-y_Br_y_)_3_ perovskite composition; however, the efficiency and the stability of the PSCs are still low compared with those of the market-leading silicon solar cells. In 2016, Saliba et al. (2016a) reported a cesium-containing triple-cation perovskite composition (FA_0.95-x_MA_x_Cs_0.05_Pb(I_1-y_Br_y_)_3_), which showed a significantly improved thermal stability but less δ-phase impurities than the previous perovskite compositions. The so-called triple-cation perovskite recipe was less sensitive to the fabrication conditions compared with the previous perovskite compositions. Thus the reproducibility was largely improved, making the triple-cation perovskite composition one of the most commonly used recipes up to the present date. Due to the significantly improved crystallization of the perovskite film by introducing the Cs cation, a stabilized power output of over 21% was demonstrated (Saliba et al., 2016a). Most importantly, a stabilized power output under continuous 1 sun illumination over 200 h was demonstrated for the first time, which makes the triple-cation perovskite a milestone in the field of PSCs.

Considering that the band gap of the triple-cation perovskite is as high as 1.63 eV, whereas the *V*_*OC*_ of the PSCs achieved was only ∼1.15 V, there was still a significant *V*_*OC*_ loss compared with that of the commercial silicon solar cells (Saliba et al., 2016b). So, it was very important to further reduce the *V*_*OC*_ loss to improve the overall PCE. (Saliba et al., 2016b) reported a quadruple-cation perovskite composition employing Rb cation together with the triple-cation perovskite composition. It was demonstrated that the *V*_*OC*_ loss could be dramatically decreased to 0.39 V, which is comparable to the 0.4 V *V*_*OC*_ loss for commercial silicon solar cells. As a result, a stabilized PCE of 21.6% was achieved. Most importantly, a stabilized power output under 1 sun illumination at 85°C for 500 h was demonstrated. There are also some other similar works reporting that K-doped triple-cation perovskite-based PSCs have higher PCE due to the improved *V*_*OC*_ (Bu et al., 2017; Jalebi et al., 2018).

It is noted that the high PCE achieved with alkali metal cation and Br^−^ halide doping is mainly due to the increased *V*_*OC*_, which is a result of the high-quality perovskite film. However, the high-quality film comes at the cost of a blue-shifted absorption spectrum, which limits the *J*_*SC*_ to ∼23 mA/cm^2^ and therefore the overall PCE. To improve the *J*_*SC*_, the mixed cations, especially the bromide ions, need to be removed from the perovskite composition (Turren-Cruz et al., 2018). There was a need to develop a new method to produce high-quality FA-based perovskite films without using bromide.

### FAPbI_3_-Based PSCs

Given that the *V*_*OC*_ and FF achieved so far are quite close to the Shockley-Queisser optimum through the aforementioned compositional engineering, further increasing the *J*_*SC*_ becomes the main direction. Bromide has been commonly used in the previous perovskite compositions to improve the perovskite film quality; however, this comes at the cost of less-visible absorptions. Furthermore, bromide can cause phase segregations under long-term illuminations, which hinders the long-term stability of the PSCs. Thus, bromide-free FA-based perovskite composition could be a possible direction for the perovskites.

Jiang et al. (2019) reported that stable non-bromide FA_0.92_MA_0.08_PbI_3_ perovskite film could be achieved with a sequential deposition method. They reported an increase of ∼1 mA/cm^2^ *J*_*SC*_ by reducing the bromide content in the perovskite composition, and a certified efficiency of 23.3% was achieved for planar structure PSCs. In addition to the bromide content, MA was also found to be unsuitable for long-term stability due to the volatile nature of MA. Turren-Cruz et al. (2018) obtained highly crystallized MABr-free perovskite films by using the alkali cations. They demonstrated FA_0.85_Cs_0.1_Rb_0.05_PbI_3_-based PSCs with superior stability under maximum power point (MPP) tracking for 1,000 h. There have been some other reports that highly crystallized pure α-phase FAPbI_3_ film can be achieved using MACl as an additive, which could be re-evaporated during the annealing process (Kim et al., 2019; Min et al., 2019). Kim et al., 2019) reported that mixing 40% MACl with FAPbI_3_ could largely improve the crystallinity and grain size of the final FAPbI_3_ perovskite film. Due to the volatile nature of MACl, the resulting perovskite film had very similar absorption spectra as that of pure FAPbI_3_. Thus, a *J*_*SC*_ as high as 25.6 mA/cm^2^ and a certified efficiency of 23.48% was achieved. Similarly, Min et al. (2019) reported a *J*_*SC*_ as high as 26.7 mA/cm^2^ and a certified efficiency of 23.7% using an additional additive of methylenediammonium dichloride (MDACl_2_) together with MACl and FAPbI_3_.

We summarize that there is a clear trend of compositional change from the MAPbI_3_ to FAPbI_3_ perovskite used for the record high-efficiency PSCs. Unfortunately, a direct cation substitution of MA in MAPbI_3_ with FA cannot form stable FAPbI_3_. Therefore, there have been lots of compositional engineering works about forming and stabilizing highly crystallized α-phase FAPbI_3_. Initially, various kinds of mixtures including MA, alkali metals, Br, etc., were used to improve the performance of FA-based PSCs. Later, these mixtures were excluded gradually to maximize the *J*_*SC*_ of the FA-based PSCs. Figure 3B simply illustrates the compositional development process. Interestingly, the PCE of the PSCs has been increasing obviously with the compositional changes. Figure 3C illustrates the PCE change with the perovskite compositions.

## Interface Engineering of PSCs

Compositional engineering of the perovskite absorber layer has increased the efficiency of PSCs by maximizing both the *J*_*SC*_ and *V*_*OC*_ as discussed in earlier sections. However, when the perovskite composition was close to FAPbI_3_, there was not much improvement regarding the *J*_*SC*_, besides mixing lead with tin, which so far suffers from low device efficiencies and poor stability. Meanwhile, a lot of attention has been paid to the interface engineering of the PSCs. Historically, the surface chemistry has been important for the semiconductors such as Si and GaAs for the development of solar cell techniques (Aberle, 2000; Bertness et al., 1994; Sheldon et al., 2012). It is well known that a high density of crystallographic defects could exist on the surface of the perovskite films, as perovskite films are made mainly from a solution-based technique. Nowadays, high-quality polycrystalline perovskite films that have monolithic grains can be realized where the bulk defects are negligible compared with the surface defects. Thus, interface engineering plays a more and more important role in the PSCs toward the record high efficiency and stability.

**Figure 4:**
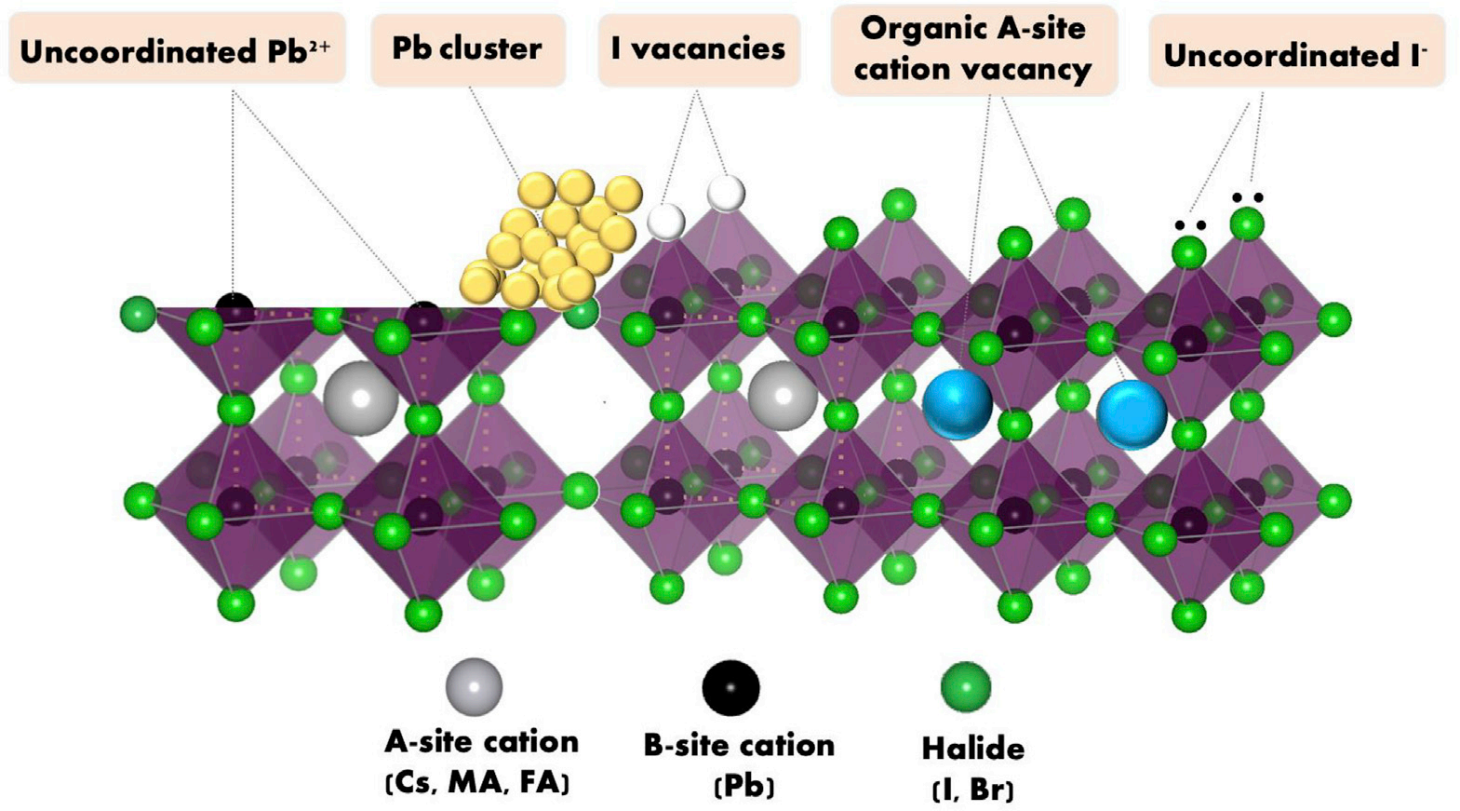
The Possible Surface Defects of Perovskite, Including Uncoordinated Pb^2+^ Ions, Pb Cluster, Iodide Vacancies, Organic A Cation Vacancy, and Uncoordinated I^−^ ions.

The study of surface passivation of the PSCs can go back to the discovery that excess PbI_2_ can promote the efficiency of PSCs by reducing carrier recombination in the perovskite layer, as well as in the interfaces between perovskite absorber and electron/hole-transporter layers (Chen et al., 2014). Since then, excess PbI_2_ has been commonly used for the perovskite compositions (Saliba et al., 2016a, 2016b; Turren-Cruz et al., 2018). Later, Bi et al. showed a record reduction of *V*_*OC*_ loss and external electroluminescence quantum efficiency for the PSCs by carefully tuning the amount of PbI_2_ in the perovskite composition (Bi et al., 2016). We know that the perovskite surface could contain a few kinds of defects, such as under-coordinated Pb^2+^ ions, Pb cluster, iodine vacancies, organic A cation vacancy, and under-coordinated I^−^ ions (Figure 4). These surface defects can result in charge recombination via non-radiative channels and thus impair device performance and lead to fast degradation of the PSCs. Interface engineering could be an effective method to reduce these unproductive defects as the ionic nature of the perovskite lattice enables molecular passivation. There have been a lot of molecules reported for perovskite surface passivation so far, and the functional groups have been studied in depth. In this review, we will go through some widely reported passivation strategies.

### Passivation via Low-Dimensional Perovskites

**Figure 5:**
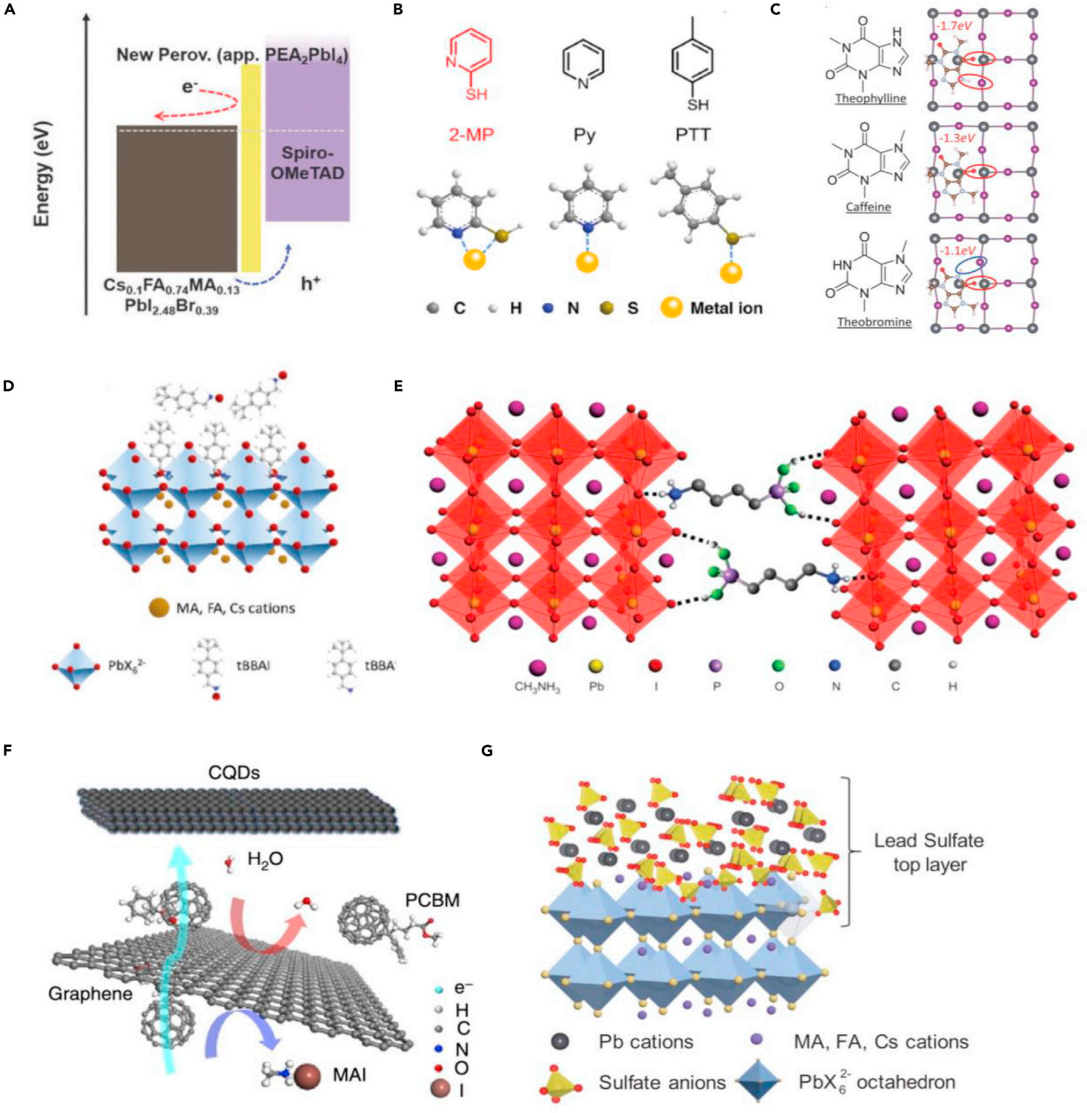
Passivation Strategies of PSCs (A) A description of how the 2D perovskite capping layer improved the PCE. At the rear region of the perovskite film, the 2D perovskite having a wide band gap can block transfer or excited electrons to the HTM layer. Reproduced with permission (Cho et al., 2017). Copyright 2017, Royal Society of Chemistry. (B) Molecular structure of passivating materials and schematic illustration of their interaction with metal ions. Reproduced with permission (Zhang et al., 2019). Copyright 2019, Wiley-VCH. (C) Theoretical models of perovskite with molecular surface passivation of Pb_I_ anti-site with theophylline, caffeine, and theobromine. Reproduced with permission (Wang et al., 2019). Copyright 2019, American Association for the Advancement of Science. (D) A simplified diagram representing a tBBAI-passivated perovskite surface. Reproduced with permission (Zhu et al., 2020). Copyright 2020, Wiley-VCH. (E) Schematic illustration of two neighboring grain structures cross-linked by butylphosphonic acid 4-ammonium chloride hydrogen bonding interactions. Reproduced with permission (Li et al., 2015). Copyright 2015, Nature Publishing Group. (F) Schematic drawing of the diffusion processes within the nanocarbon-based ETL. Reproduced with permission (Bi et al., 2017). Copyright 2017, Nature Publishing Group. (G) Schematic illustration of protection of perovskites through *in situ* formation of a lead sulfate top layer on the perovskite surface. Reproduced with permission (Yang et al., 2019). Copyright 2019, American Association for the Advancement of Science.

Low-dimensional perovskite, such as two-dimnesonal (2D) perovskite, has shown superior stability and water resistance compared with the 3D counterparts, which is due to the long-chain organic cations of the 2D perovskite. However, 2D PSCs usually show a low PCE, as the long-chain organic cations act as insulating spacing layers, which inhibit the out-of-plane charge transport (Tsai et al., 2016). To combine the advantages of the 2D perovskite with normal 3D PSCs, Grancini et al. (2016) and Cho et al. (2017) reported a 3D/2D interface engineering work growing a thin 2D perovskite layer on top of the bulk 3D perovskites. In the novel 3D/2D perovskite system reported by Cho et al., the 2D perovskite layer (PEA_2_PbI_4_ [PEA = phenylethylammonium]) acted as a protecting layer against ion migrations and environmental moistures (Cho et al., 2017). Interestingly, the large band-gap 2D perovskite could energetically wrap grains of the narrow band-gap 3D perovskite, establishing a heterojunction at the grain boundaries, which facilitated the hole transportation (Figure 5A) (Cho et al., 2017). Moreover, the conversion of defect-rich grain boundaries/surface into 2D perovskite might also healed these defects to dramatically reduce defect density. In the end, highly stable and efficient 3D/2D PSCs could be achieved (Grancini et al., 2016; Cho et al., 2017).

To further improve the 3D/2D perovskite interface engineering, the electrical properties of the 2D structure and resistance to the moisture must be understood. Kim et al. (2019) reported a systematic interfacial study using several long-chain alkylammonium iodides, including butylammonium iodide, octylammonium iodide (OAI), and dodecylammonium iodide, to form a 2D structure on top of the bulk 3D perovskite. It was found that the length of the alkyl chain played an important role in the electron-blocking ability and humidity resistance. By using post-treatment of OAI, a certified efficiency of 22.9% was achieved (Kim et al., 2019). In addition to the length of the alkyl chains, Zhang et al. (2019) reported that by fluorine substitution on the para position in PEA to form a 4-fluorophenethylammonium, the average phenyl ring centroid-centroid distances became shorter with better aligned stacking of perovskite sheets. Thus, the orbital interactions, as well as the charge transport across adjacent inorganic layers, could be improved. Liu et al. (2019) further demonstrated an ultrahydrophobic 3D/2D water-resistant PSC with efficiencies over 22% using pentafluorophenylethylammonium as a fluoroarene cation inserted between the 3D light-harvesting perovskite film and the HTM.

### Passivation via Lewis Bases

Lewis base passivation has been demonstrated to be another important interface engineering strategy. It is well known that Lewis bases can donate a pair of non-bonding electrons to coordinate with, and passivate, the under-coordinated Pb^2+^ or I (iodine) vacancies, forming a Lewis adduct. Noel et al. (2014) first reported a Lewis base surface treatment by depositing a thin layer of thiophene and pyridine on top of the perovskite surface. The strong coordinate bonding between the sulfur atom in thiophene or nitrogen atom in pyridine with under-coordinated Pb^2+^ can effectively passivate the Pb^2+^ point defects. Upon this Lewis base treatment, a dramatic enhancement in the time-resolved photoluminescence (TRPL) lifetime of the perovskite films and improved stability for the MPP measurement were observed. Similarly, Yavari et al. (2018) demonstrated that the use of poly(4-vinylpyridine) (PVP), a polymer functionalized with pyridine, as a passivation agent could improve the performance of the MAPbI_3_-based PSCs.

Other Lewis bases containing amines (such as –NH_2_) have also shown effective passivation of the PSCs. For example, Wang et al. (2016) illustrated that spin coating a diluted solution of phenylalkylamines on the perovskite surface could effectively passivate the surface defects. Most impressively they reported an increase in PCE from 14.2% to 19.2% for the FAPbI_3_-based PSCs passivated by benzylamine. The benzylamine passivated device showed longer carrier lifetime and improved diode characteristics, which should be due to the reduction of trap states related to surface defects. Furthermore, Lin et al. employed π-conjugated small molecules, indacenodithiophene end capped with 1.1-dicyanomethylene3-indanone, to passivate the surface traps of the hybrid perovskites (Lin et al., 2017). The π-conjugated Lewis base is essentially a type of organic semiconducting molecules attached with Lewis base blocks. The Lewis base (carbonyl [C=O] and cyano [C=N]) can effectively passivate the traps on the surface or at the grain boundaries (e.g., under-coordinated Pb^2+^ ions and Pb clusters), and the n-type π-conjugated materials possess chemical properties that promote electron extraction and electron transport. Such molecules combine the multifunction that can combine trap passivation and charge extraction.

Another example of Lewis base passivation is the use of oxygen-containing alkylphosphine oxides as well as phosphorus of alkylphosphines to passivate the surface defects of the perovskite layer. deQuilettes et al. (2016) systematically investigated three Lewis bases, including trio-n-octylphosphine oxide (TOPO), 1-octadecanethiol, and triphenylphosphine (PPh_3_), and demonstrated that TOPO treatment increased the photoluminescence quantum yield (PLQY) and the TRPL lifetime of the perovskite film the most. These results clearly showed the role of phosphorus and oxygen functionalities in reducing the non-radiative recombination. It must be noted that the alkylphosphine oxides and/or alkylphosphine passivation layer could be washed out by chlorobenzene or 2-proponal, which indicated a weak bonding between these molecules and the perovskite surface.

To solve the problem that most passivating agents are weakly anchored with the perovskite surface, Zhang et al. (2019) reported a bidentate molecule, 2-mercaptopyridine (2-MP), to increase anchoring strength for improving the passivation efficacy and stability. 2-MP has a nitrogen atom in pyridine ring and a sulfur atom in a mercapto group (Figure 5B), both of which can coordinate with Pb^2+^, thus substantially improving the binding strength and adhesion of the passivation layer on the perovskite surface. Compared with the monodentate counterparts of pyridine and toluenethiol (Figure 5B), 2-MP passivation on a MAPbI_3_ film resulted in a 2-fold improvement of TRPL lifetime and remarkably enhanced tolerance to chlorobenzene washing and vacuum heating, which improved the PCE of the PSCs from 18.35% to 20.28%, with open-circuit voltage approaching 1.18V. Moreover, the MAPbI_3_ films passivated with 2-MP exhibited unprecedented humid-stability.

In addition to the Lewis base functional groups, molecular configuration can also play an important role, especially when multifunctional groups are employed. Recently, to acquire an in-depth understanding of how the chemical environment of a functional group influences the passivation effectiveness, Wang et al. (2019) systematically investigated the surface defect passivation by incorporating a set of tailored small molecules, namely, theophylline, caffeine, and theobromine, shown in Figure 5C, interacting with the surface defects. It was found that when N-H and C=O were in an optimal configuration in the molecule, hydrogen-bond formation between N-H and I (iodine) assisted the primary C=O binding with the anti-site Pb defect to maximize surface-defect binding. A stabilized PCE of 22.6% of PSC was demonstrated with theophylline treatment. Also, theophylline-treated PSCs showed significant improvement in the stability.

### Passivation via Organic Ammonium Halide Salts

Organic ammonium salts with functional groups of ammonium (–NH_3_^+^) have also been shown as effective passivation agents. Unlike amines (–NH_2_), which have a lone pair of electrons capable of coordinating to positively charged Lewis acid defects via coordinative bonding, the –NH_3_^+^ cations usually passivate negatively charged defects through electrostatic interactions including ionic bonding and hydrogen bonding. Zhao et al. (2016) reported that surface passivation of MAPbI_3_ with a linear diammonium iodide (NH_3_(CH_2_)_8_NH_3_I) can largely reduce the surface trap defects and improve the performance of the PSCs, whereas a 3D to 2D perovskite phase transformation was induced when NH_3_(CH_2_)_4_NH_3_I was used for surface passivation, showing no improvements. Similar work reported by Jiang et al. (2019) has also shown excellent surface passivation of FA_0.92_MA_0.08_PbI_3_ perovskite with the PEAI salt, and a certified efficiency of 23.32% was achieved. Interestingly, this post-treatment cannot be followed by further annealing, as the passivation effect would be lost due to the formation of a 2D perovskite phase.

Recently, Zhu et al. (2020) reported a tailored amphiphilic molecule, 4-*tert*-butyl-benzylammonium iodide (tBBAI), shown in Figure 5D, as an efficient surface passivation material of perovskites and achieved an efficiency of 23.5% for the treated PSCs. Compared with the PEAI-based treatments, surface treatment with tBBAI showed better charge extraction from the perovskite layer to the 2,2′,7,7′-tetrakis(*N*,*N*-di-*p*-methoxyphenylamine)-9,9′-spirobifluorene (spiro-OMeTAD) hole transporter. This was due to the *tert*-butyl groups that prevented the molecular aggregations on the perovskite surface. However, the details of the passivation mechanism need further studies, even though it appears that the hydrophobic capping layer (umbrella) formed by the *tert*-butyl groups of tBBAI could protect the perovskite and facilitate wetting by the spiro-MeOTAD hole conductor film.

We note that Li et al. (2015) investigated similar ammonium salts as dopants in the perovskite film in 2015. It was found that alkylphosphonic acid ω-ammonium chlorides can hydrogen bond with iodine atoms at the surface (Figure 5E) of perovskite to improve grain cohesivity, device performance, and long-term device stability. However, further in-depth understanding of the perovskite surface passivation via organic ammonium salt will be needed in the future.

### Passivation via Thin Protective Capping Layer

Different from the above-mentioned passivation strategies, Bi et al. (2017) provided a strategy using a nanostructured carbon layer, containing N-doped graphene, the fullerene carbon quantum dots, between the perovskite layer and the Ag electrode in inverted structure PSCs (Figure 5F). The nanostructure carbon layer can effectively block the ion/molecule diffusion, which has been reported as a serious device degradation or defects generation process causing poor device stability. Meanwhile, the nanostructured carbon layer enabled a compact electron transporting layer (ETL) with perovskite, which assists electron transport while impairing penetration of ions or water. Thus, both the efficiency and stability of the nanostructured carbon layer containing PSCs were improved (Bi et al., 2017). Similarly, Arora et al. (2017) reported that the graphene interlayer could be used between perovskite and copper thiocyanate (CuSCN) hole transporter and achieved long-term stability under heat stress.

Recently, Wang et al. (2019) reported a new strategy using a chlorinated graphene oxide between perovskite and poly(triarylamine) (PTAA) hole transporter and demonstrated that the modified PSCs could maintain 90% of the initial efficiency after an operation at the MPP under AM 1.5G solar light at 60°C for 1,000 h. Compared with the previous strategies where the graphene was directly doped in ETL (Bi et al., 2017), strong Pb-Cl and Pb-O bonds formed between the perovskite film with a Pb-rich surface and a chlorinated graphene oxide layer. The constructed heterostructures can selectively extract photogenerated charge carriers and impede the loss of decomposed components from soft perovskites, thereby reducing damage to the organic charge-transporting semiconductors.

Instead of using graphene-based thin capping layers, Yang et al. (2019) reported a passivation strategy for lead halide perovskites by forming a water-insoluble lead oxysalt layer on the perovskite surface (Figure 5G) through *in situ* reaction with sulfate or phosphate ions. The capping lead oxysalt thin layers formed strong chemical bonds with the perovskite and enhanced resistance to the moisture. The wide-band-gap lead oxysalt layers also reduced the defect density on the perovskite surface by passivating the under-coordinated surface lead centers, which are defect-nucleating sites. Formation of the lead oxysalts increased the device efficiency to 21.1% and helped to maintain 96.8% of the initial efficiency after operation at MPP under simulated AM 1.5G irradiation for 1,200 h at 65°C. Similarly, Singh et al. (2018) demonstrated that sulfate anion can chemically bind to TiO_2_ surface from -Ti-O-S-. The sulfate anion works as a bridge between Pb^2+^ and TiO_2_, facilitating the electron extraction, leading to a stable efficiency of 21%.

## Conclusion and Future Directions of Highly Efficient and Stable PSCs

In conclusion, we present a compact review study of the compositional engineering of the organic-inorganic lead halide perovskite from MAPbI_3_ to FAPbI_3_. Unfortunately, direct substitution of the MA in MAPbI_3_ with FA cannot get stable pure α-phase FAPbI_3_. Therefore, various kinds of cations or halides, including MA, Cs, Br, Cl, and so on, are doped or mixed with FAPbI_3_ precursor to stabilize the final α-phase FAPbI_3_ perovskite films, achieving high efficiencies of the corresponding PSCs. However, the absorption spectrum of the mixed FAPbI_3_ is blue-shifted compared with that of pure FAPbI_3_, which limits the potential current density of the PSCs. Moreover, the mixed cations or halides can cause non-uniform perovskite films and phase segregations, which limit the long-term stabilities. Recently, highly crystallized and stable α-phase FAPbI_3_ could be achieved by mixing volatile MACl, leaving the final perovskite composition very close to the pure α-phase FAPbI_3_. In the end, highly efficient and stable FAPbI_3_-based PSCs have been demonstrated.

Regarding the Pb-based organic-inorganic hybrid perovskite composition, there is little room to further optimize the band-gap energy as the perovskite composition achieved so far has been very close to that of the pure α-phase FAPbI_3_; however, there are still some interesting directions. For example, it will be interesting to know whether pure α-phase FAPbI_3_ perovskite could be formed without using any MA additive or even any additive. We think this could happen, but it needs more understanding of the α-phase transition, especially below the thermodynamic transition temperature. With the fundamental insights obtained in compositional engineering for Pb-based perovskite, will it be possible to prepare highly efficient and stable Sn/Pb mixtures? If high-quality Sn/Pb-based perovskite can be achieved, the band gap could be pushed closer to the Shockley-Queisser optimum. It will be also very interesting to explore what perovskite composition could potentially be used for the final commercialization. We comment that pure iodide composition seems to be necessary to avoid the phase segregation under long-term illumination, and MA it seems should not be included due to its volatile nature according to the current studies. It sounds like FAPbI_3_ is a good choice for highly efficient and stable PSCs. However, some more engineering work should be explored to maximize the stability of FAPbI_3_ perovskite to reach a goal that PSCs can be used for 30 years. For example, some long-chain ligands with functional groups have been demonstrated to be promising. We also note that CsPbI_3_ could also be a potential candidate due to its excellent thermal stability.

We also present a brief summary of the interface passivation strategies of the PSCs, including the 3D/2D interface engineering, Lewis base passivation, organic ammonium salt passivation, and capping thin layer passivation. All these passivation techniques have shown effective improvements of the stability and performance of the PSCs. However, a deep understanding of the passivation mechanism, such as the organic ammonium salt passivation, is still missing. In the future, more attention should be paid to these fundamental mechanisms, which are instrumental for the rational design of the molecules with multifunctional groups toward highly efficient and stable PSCs. We believe that understanding the nature of the defects and their concentration and distribution for the different composition of perovskites can shed some light on the design and mechanism of the passivation molecules. A multilateral approach of combining computational science along with an array of experimental techniques and machine learning for the creation of a molecular library should be employed to get some more insights into the passivation mechanism. Such insights into fundamental mechanisms can lead to a more rational design by establishing a structure-property relationship. Currently, although we cannot predict which kind of interface passivation can be the best in the end, as we noted a lot of more fundamental understanding is still missing, we can conclude that a molecule with multi-functional groups that can passivate the different kinds of defects could be the direction for designing new passivation materials. Also, the passivation materials should not be limited to the as-mentioned organic molecules or salts. Rather, functionalized inorganic materials such as graphene oxide or other water-insoluble oxide layers should be explored more. By analysis of accumulated data and increasing the understanding, passivation can lead to a significant improvement in performance and stability of PSCs and push them toward commercialization.

### Limitations of the Study

In this compact review, we only focus on the organic-inorganic lead halide perovskites. There are also some other perovskite compositions such as all-inorganic lead halide perovskites and tin-based perovskites, which may show different results from what we present in this review.

### Resource Availability

#### Lead Contact

Further information and requests for resources should be directly to and will be fulfilled by the Lead Contact, Haizhou Lu (haizhou.lu@epfl.ch).

#### Materials Availability

This study did not generate any new unique reagents.

#### Data and Code Availability

The data that support the findings of this study are available from the correspinding anuthor on reasonable request.
